# Lipopolysaccharide of *Legionella pneumophila* Serogroup 1 Facilitates Interaction with Host Cells

**DOI:** 10.3390/ijms241914602

**Published:** 2023-09-27

**Authors:** Bożena Kowalczyk, Markus Petzold, Zbigniew Kaczyński, Agnieszka Szuster-Ciesielska, Rafał Luchowski, Wiesław I. Gruszecki, Beate Fuchs, Christina E. Galuska, Adam Choma, Jacek Tarasiuk, Marta Palusińska-Szysz

**Affiliations:** 1 Department of Genetics and Microbiology, Institute of Biological Sciences, Faculty of Biology and Biotechnology, Maria Curie-Sklodowska University, 20-033 Lublin, Poland; b.kowalczyk746@wp.pl (B.K.); adam.choma@mail.umcs.pl (A.C.); jacek.tarasiuk@mail.umcs.pl (J.T.); 2 Institute of Medical Microbiology and Virology, University Hospital Carl Gustav Carus, University of Technology Dresden, 01069 Dresden, Germany; markus.petzold@ukdd.de; 3 Laboratory of Structural Biochemistry, Faculty of Chemistry, University of Gdansk, 80-309 Gdansk, Poland; zbigniew.kaczynski@ug.edu.pl; 4 Department of Virology and Immunology, Institute of Biological Sciences, Faculty of Biology and Biotechnology, Maria Curie-Sklodowska University, 20-033 Lublin, Poland; agnieszka.szuster-ciesielska@mail.umcs.pl; 5 Department of Biophysics, Institute of Physics, Faculty of Mathematics, Physics and Computer Science, Maria Curie-Sklodowska University, 20-031 Lublin, Poland; rafal.luchowski@mail.umcs.pl (R.L.); wieslaw.gruszecki@mail.umcs.pl (W.I.G.); 6 Research Institute for Farm Animal Biology (FBN), Core Facility Metabolomics, 18196 Dummerstorf, Germany; fuchs.beate@fbn-dummerstorf.de (B.F.); galuska.christina@fbn-dummerstorf.de (C.E.G.)

**Keywords:** legionnaires’ disease, lipopolysaccharide, lipids, TNF-α, IL-6

## Abstract

*Legionella pneumophila* is the primary causative agent of Legionnaires’ disease. The mutant-type strain interrupted in the ORF7 gene region responsible for the lipopolysaccharide biosynthesis of the *L. pneumophila* strain Heysham-1, lacking the *O*-acetyl groups attached to the rhamnose of the core part, showed a higher surface polarity compared with the wild-type strain. The measurement of excitation energy transfer between fluorophores located on the surface of bacteria and eukaryotic cells showed that, at an early stage of interaction with host cells, the mutant exhibited weaker interactions with *Acanthamoeba castellanii* cells and THP-1-derived macrophages. The mutant displayed reduced adherence to macrophages but enhanced adherence to *A. castellanii*, suggesting that the *O*-acetyl group of the LPS core region plays a crucial role in facilitating interaction with macrophages. The lack of core rhamnose *O*-acetyl groups made it easier for the bacteria to multiply in amoebae and macrophages. The mutant induced TNF-α production more strongly compared with the wild-type strain. The mutant synthesized twice as many ceramides Cer(t34:0) and Cer(t38:0) than the wild-type strain. The study showed that the internal sugars of the LPS core region of *L. pneumophila* sg 1 can interact with eukaryotic cell surface receptors and mediate in contacting and attaching bacteria to host cells as well as modulating the immune response to infection.

## 1. Introduction

*Legionella* spp. are facultative aerobic bacteria that primarily thrive in freshwater environments and artificial water systems. They are commonly found as an essential part of intricate biofilm structures attached to surfaces in aquatic settings. Various species of protozoa, including those from the genera *Acanthamoeba*, *Naegleria*, and *Tetrahymena*, play a crucial role in the ecology and pathogenesis of *Legionella*, facilitating the intensive multiplication of the bacteria [1]. The proliferation of *Legionella* within protozoan cells is possibly due to the use of numerous cell biology tools by the bacteria [2,3]. Host cell manipulation tools that evolved in interaction with protozoa have allowed *Legionella* to infect lung macrophages, becoming a human pathogen. The bacteria cause a severe pneumonia known as Legionnaires’ disease, which can be fatal if not diagnosed and treated promptly.

Currently, there are 72 known species within the genus *Legionella*, although not all of them contribute equally to the laboratory-confirmed cases of Legionnaires’ disease worldwide [4]. Among these species, *L. pneumophila* is the predominant cause, responsible for approximately 80–90% of cases in Europe and the United States [5]. Moreover, even within the *L. pneumophila* species, the distribution of disease-causing strains is uneven, with serogroup (sg) 1 strains accounting for around 90% of cases [6], although *L. pneumophila* sg 2–15 is more common in building water systems than *L. pneumophila* sg 1 [7,8].

The serogroup of *L. pneumophila* is determined by the composition of its lipopolysaccharide (LPS), which serves as a structural component and significantly influences the pathogen’s properties and capabilities. LPS is a glycolipid consisting of a surface-exposed O-specific chain, a core oligosaccharide divided into an outer and an inner part, and a lipid A, which anchors the entire LPS molecules to the outer membrane of the bacterial cell envelope. The lipid A sugar backbone of *L. pneumophila* LPS is composed of a phosphorylated disaccharide consisting of 2,3-diamino-2,3-dideoxy-d-glucose (GlcN3N). The GlcN3N subunits are linked by amide bonds with hydroxy fatty acids, which are acylated by linear, branched (*iso* or *anteiso*), and long-chain fatty acids [9,10]. Fatty acids [28:0 (27-oxo) and 27-dioic], possessing the double length as highly potent enterobacterial acyl groups, may be responsible for the low endotoxicity of *L. pneumophila* lipid A. The outer part of the oligosaccharide core consists of rhamnose (Rha), mannose (Man), acetylquinovosamine (QuiNAc), and acetylglucosamine (GlcNAc) in the molar ratio of 2.1:1.1:1:1.4 [11,12]. The presence of *N*- and *O*-acetyl groups of amino sugars (QuiNAc and GlcNAc) and deoxy sugars (Rha and QuiNAc) impart a distinct hydrophobic character to the outer core. The inner core contains two 3-deoxy-d-manno-2-octulosonic (Kdo) acid molecules bound by a 2→4 ketosidic linkage. The distal Kdo is decorated by d-mannose connected to the C8 position. The hydrophilic inner core of *L. pneumophila* LPS exhibits similarities and differences compared with enterobacterial core oligosaccharides in containing Kdo but lacking heptose and phosphate groups. The O-specific chain of the *L. pneumophila* Philadelphia strain is a homopolymer containing 10 to 75 repeat units of 5-acetamidino-7-acetamido-8-*O*-acetyl-3,5,7,9-tetradeoxy-l-glycero-d-galacto-non-2-ulosonic acid (legionaminic acid) [13]. High heterogeneity within the O-specific chain of LPS provided the basis for the classification of *L. pneumophila* into 15 serogroups. The application of monoclonal antibodies (mAbs) from the Dresden panel, which recognize specific LPS epitopes, facilitated the division of sg 1 strains into nine distinct subgroups. These subgroups were designated as mAb 3/1-positive, Pontiac strains (Philadelphia, Knoxville, Benidorm, France/Allentown) and mAb 3/1-negative, non-Pontiac strains (Bellingham, Oxford, OLDA, Heysham, Camperdown) [14]. The epitope recognized by the mAb3/1 antibody is the 8-*O*-acetyl group of polylegionaminic acid, transferred by the *O*-acetyltransferase encoded by the *lag-1* gene. However, the *O*-acetylation pattern of the *L. pneumophila* sg 1 core oligosaccharide and short-chain O-polysaccharide is *lag-1*-independent [15].

The genetic background of LPS molecules in *L. pneumophila* sg 1 strains consists of a highly conserved 15 kb region and an sg1-specific, variable 18 kb region, which is partially disrupted by phage-related genes. The region spanning from ORF6 to ORF11 of the sg 1-specific region is likely involved in late LPS modification [16].

The presence of *O*-acetyl LPS groups in *L. pneumophila* sg 1 contributes to an increase in the hydrophobicity of the bacterial surface. This characteristic plays a crucial biological role both in the dissemination of bacteria by facilitating the formation of contaminated aerosols and in interacting with host cells at various stages, including adhesion to protozoa and macrophage cells, as well as promoting intracellular survival by inhibiting complement-mediated lysis and neutrophil phagocytosis [17]. Epidemiological studies have confirmed a direct correlation between the epitope recognized by mAb3/1 and the increased virulence of *L. pneumophila* sg 1. The community- and nosocomial-acquired, as well as travel-associated isolates from patients with Legionnaires’ disease, were predominantly recognized by this antibody [14]. However, the proportion of mAb 3/1-negative sg 1 isolates was significantly higher in nosocomial cases compared with community- or travel-associated cases [14]. Moreover, the ORF7 gene biosynthesis LPS was harbored by significantly more of the clinical isolates of *L. pneumophila* sg1 compared with the environmental isolates in Japan and China [18].

To investigate how modifications of the LPS core region influence interactions with host cells and the modulation of the immune response, the Heysham-1 strain of *L. pneumophila* sg1, belonging to the non-Pontiac group, and the mutant interrupted in the ORF7 gene region were employed.

## 2. Results

### 2.1. Phenotypical Screening

**Figure 1:**
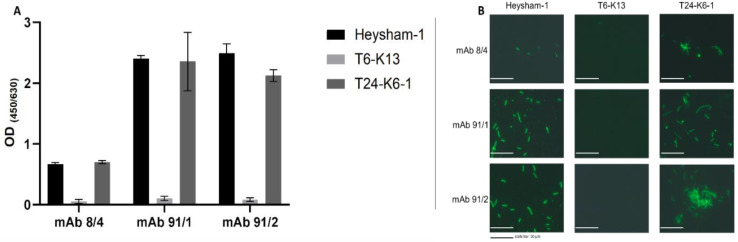
Reactivity of Heysham-1 (wild-type), T6-K13 (mutant-type), and T24-K6-1 (complementant) strains with monoclonal antibodies using (**A**) indirect ELISA (four experimental repeats indicated with standard deviation) and (**B**) indirect immunofluorescent assay. Scale bar = 10 µm.

The reactivity of the wild-type *L. pneumophila* Heysham-1 strain, the mutant-type T6-K13 strain, and the complementant T24-K6-1 strain with the mAb antibodies showed an altered phenotype demonstrated as the loss of reactivity with mAb 91/1 and 91/2 (both IgG3, κ) in ELISA and indirect immunofluorescence. These mAbs are part of the extended Dresden panel (BALB/c mice immunized with the *L. pneumophila* strain Corby) [19,20]. The complementant T24-K6-1 strain restored this phenotype (Figure 1). There is no difference between the wild-type, mutant-type, and complementant strains regarding the growth behavior and the colony morphology, color, and texture.

### 2.2. NMR Spectroscopy

**Figure 2:**
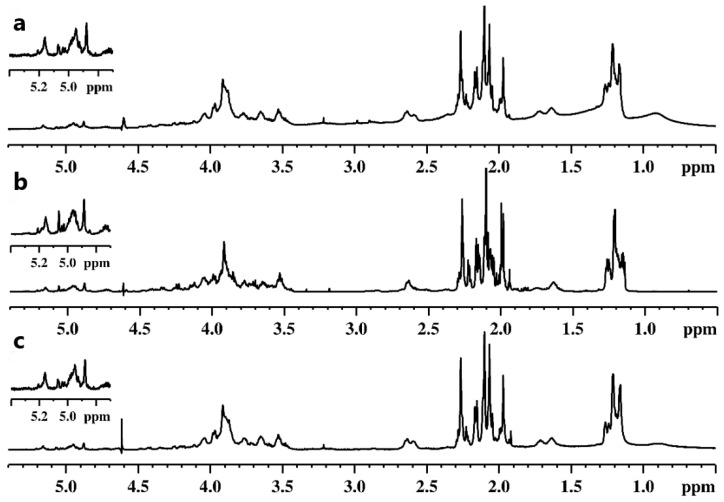
^1^H NMR spectra (39 °C, 700 MHz) of *Legionella* the O-polysaccharides: (**a**) Heysham-1, (**b**) T6-K13, (**c**) T24-K6-1. The enlargement of the anomeric region is placed in the upper left corner of each spectrum.

Three different OPSs of *Legionella* (Heysham-1, T6-K13, and T24-K6-1) were analysed using NMR spectroscopy. The profiles of ^1^H NMR spectra were very similar (Figure 2).

**Figure 3:**
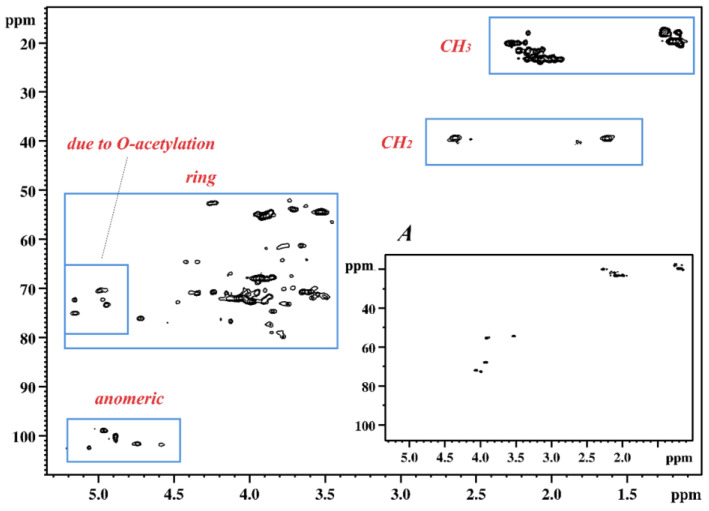
The HSQC NMR spectrum (39 °C, 700 MHz) of the OPS of the *L. pneumophila* T6-K13 strain. The blue frames group cross-peaks from similar ^1^H–^13^C arrangements. Graph A: The spectrum in the lower right corner shows only cross-peaks from high-intensity proton signals on the ^1^H NMR spectrum (threshold shifted upward).

The spectra showed proton signals in ranges characteristic of polysaccharides and their non-sugar substituents: anomeric protons (δ 4.5–5.2), sugar ring protons (δ 3.4–4.5), and methyl protons of deoxy sugars (δ 1.1–1.3) as well as methyl protons of acetyl groups (δ 1.9–2.4). Preliminary analysis of ^1^H NMR spectra in the anomeric range revealed a high heterogeneity of the polysaccharides, which was evidenced by a large number of signals with different intensities and widths. This observation was also confirmed by a very large number of signals of various intensities in the range of so-called acetyl groups (δ 1.9–2.4). In addition, a large difference was found in the intensity of the sugar ring signals compared with those of the anomeric range. To solve this problem, it was necessary to record and analyse 2D NMR spectra. The further description was focused on sample T6-K13 due to the aforementioned similarity of the analysed samples. The HSQC spectrum confirmed the observation from the analysis of the ^1^H spectra. The characteristic areas of cross-peaks originating from ^1^H–^13^C correlation (methyl groups, methylene groups, ring, anomeric) were identified on the spectrum (Figure 3).

The analyses of only the most intensive signals on the HSQC spectrum (Figure 3A) revealed five cross-peaks in the sugar ring region at δ 4.057/72.06, δ 3.994/72.72, δ 3.920/67.91, δ 3.913/55.43, and δ 3.529/54.46, two cross-peaks of one methylene group at δ 2.635/1.630/39.51, and one cross-peaks of the methyl group at δ 1.202/19.72, as well as a few cross-peaks from methyl groups of acetyl groups. The TOCSY spectrum showed that all cross-peaks (except those of acetyl groups) belonged to one spin system. The COSY spectrum allowed the order of protons in the spin system to be determined starting from the methyl group. In addition, the HMBC spectrum was used to assign two quaternary carbon atoms C-2 and C-1, respectively, δ 101.87, and δ 174.36. The chemical shift values of all carbon atoms and protons of the OPS (Table 1) were determined based on COSY and TOCSY homonuclear spectra, as well as HSQC and HMBC heteronuclear NMR spectra.

The identification of the sugar residue was achieved through the comparison of NMR data with the literature [13,21,22,23]. The values collected in Table 1 were basically identical to those presented for the O-specific chain of *L. pneumophila* sg 1 [13]. The relatively high value of C-4 (δ 72.06) indicated the position of substitution. The OPS of the mutant of *L. pneumophila* was a homopolymer consisting of 2→4 linked 5-acetamidino-7-acetamido-3,5,7,9-tetradeoxy-l-glycero-d-galacto-nonulosonic acid residues.

A detailed analysis of the NMR spectra recorded for the Heysham-1 strain and the T24-K6-1 strain gave essentially the same chemical shift values as for the OPS of the T6-K13 strain.

According to the assigned chemical structure of OPS, the relatively small intense signals in the anomeric range of the ^1^H spectrum (Figure 2) must have originated from the core part. A significant number of cross-peaks moved to the higher chemical shift values in the proton domain of the HSQC spectrum (Figure 3) was indicative of a high degree of *O*-acetylation. This observation explained the very complex profile of the ^1^H spectrum in the anomeric range. Also, this finding is consistent with literature data on the structure of the core part of *L. pneumophila* sg 1, which was characterised by a very high degree of acetylation [11].

**Figure 4:**
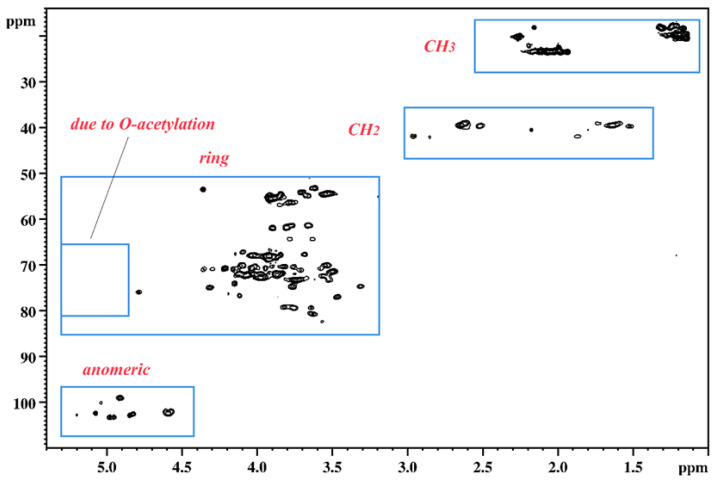
The HSQC NMR spectrum (39 °C, 700 MHz) of the de-*O*-acetylated OPS of the T6-K13 strain. The blue frames group cross-peaks from similar ^1^H-^13^C arrangements.

In the next step, an attempt was made to simplify the NMR spectra through the de-*O*-acetylation of the OPS. The HSQC spectrum recorded for the de-*O*-acetylated sample T6-K13 confirmed the effectiveness of the reaction (Figure 4).

**Figure 5:**
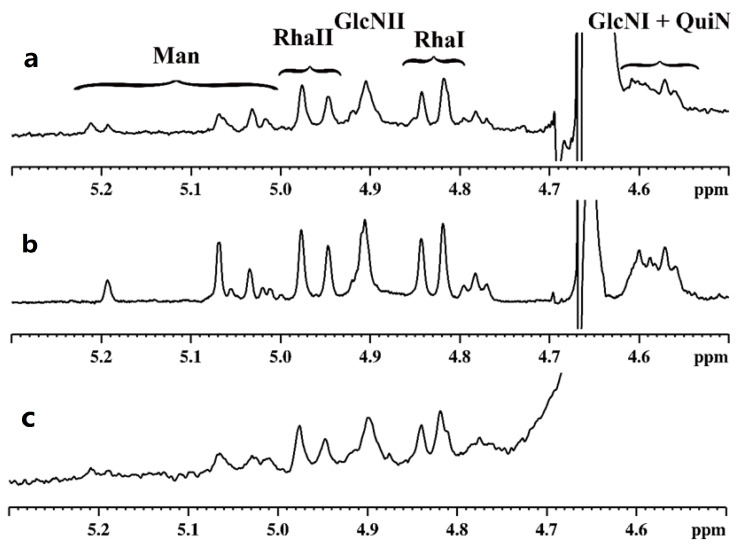
The anomeric regions of ^1^H NMR spectra (39 °C, 700 MHz) of *L. pneumophila* de*-O-*acetylated O-polysaccharides: (**a**) Heysham-1, (**b**) T6-K13, (**c**) T24-K6-1.

No cross-peaks were observed in the previously marked characteristic region. The recorded ^1^H NMR spectra for all de-*O*-acetylated samples showed very high similarity (Figure 5).

The comparison of the recorded spectra with the literature allowed a preliminary identification of the signals and a possible structure of the core part to be proposed [11].


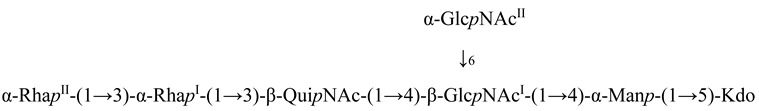


In the spectra recorded for the analysed samples, for each of two Rha residues, two anomeric signals were identified. To explain this observation, it would be necessary to construct an R-LPS-producing mutant.

A detailed comparison showed that the Heysham-1 and T24-K6-1 spectra are virtually identical (Figure 5). However, they differ slightly from T6-K13 in the range of the anomeric proton signals. A large number of the anomeric signals of the mannose residue was due to the presence of the various forms of Kdo residue to which the mannose residue is attached.

### 2.3. A Mutation in the LPS Synthesis Gene Affects the Lipid Profile of L. pneumophila

**Figure 6:**
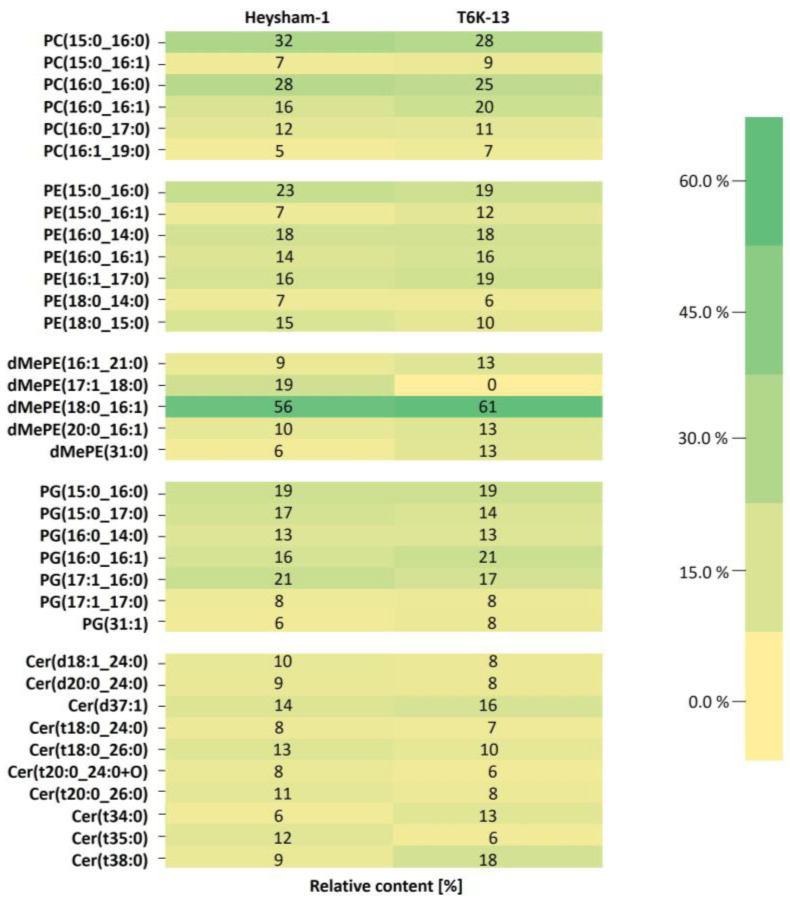
Lipidome heatmap. Relative abundance of the main lipid species of the *L. pneumophila* Heysham-1 strain and the mutant T6-K13 strain. Phosphatidylcholines (PC), phosphatidylethanolamines (PE), dimethylphosphatidylethanolamines (dMePE), phosphatidylglycerols (PG), and ceramides (Cer) were analysed by LC-MS/MS in the positive ion mode.

Lipidomic analysis based on liquid chromatography–mass spectrometry (LC-MS) has allowed for the comprehensive identification of approx. two hundred lipid molecular species in the *L. pneumophila* strains. The lipid identified in the *L. pneumophila* Heysham-1 strain and the mutant-type T6K-13 strain included triglycerides (TG), diglycerides (DG), phosphatidylcholines (PC), phosphatidylethanolamines (PE), dimethylphosphatidylethanolamines (dMePE), phosphatidylglycerols (PG), cardiolipins (CL), and ceramides (Cer). Lipidomic analyses showed no significant differences in the content of individual lipid classes among the tested strains. However, lipidomic profiling revealed differences in the content of molecular species between the *L. pneumophila* strains. The main lipid fractions of the Heysham-1 strain and the mutant-type strain were phospholipids, dominated by phosphatidylcholine (PC) and phosphatidylethanolamine (PE). A structural analysis of phospholipids (PLs) showed that all the tested strains synthesized 23 different PCs with fatty acids with 14 to 22 acyl chain lengths, saturated and monounsaturated, and fatty acid with a cyclopropyl ring (cyclopropyl 17:0). The main phosphatidylcholines were PC(15:0_16:0), PC(16:0_16:0), and PC(16:0_16:1). The wild-type strain synthesized more PC(15:0_16:0) and PC(16:0_16:0) than the mutant-type strain. The T6K-13 strain was characterized by a higher content of PC(16:0_16:1) and PC(16:1_19:0) compared with the Heysham-1 strain (Figure 6).

Molecular species PE(15:0_16:0), PE(16:1_17:0), and PE(18:0_15:0) dominated in the PE class. The Heysham-1 strain contained more PE(15:0_16:0) and PE(18:0_15:0) compared with the mutant-type strain.

The analysed strains also synthesized methylated derivatives of PE. The major species in the dMePE class was dMePE(18:0_16:1), which accounted for approx. 60% of all synthesized methylated PE derivatives by these strains. The wild-type strain produced a considerable amount of dMePE(17:1_18:0) (19%), while the T6K-13 mutant had none of this dMePE species.

A comparative analysis of the PG profile showed that the Heysham-1 strain was characterized by a higher content of PG(17:1_16:0) (21%) and PG (15:0_17:0) (17%) compared with the mutant, synthesizing 17% and 14% of these PL species, respectively. TGs were the class with the greatest structural diversity including 112 different TGs, of which TG(18:0_16:0_18:0) and TG(16:0_16:0_16:1) were dominant. The relative content of different species of the lipid class in *L. pneumophila* strains was very similar. However, the mutant-type strain was characterized by a higher production of TG(16:0_16:0_16:0) and TG(18:0_16:0_18:0), compared with the wild-type strain. In contrast, the Heysham-1 strain synthesized approx. 3% more TG(18:1_18:1_18:1) than the T6-K13 strain (Figure S1). The analysed strains synthesized 23 different DGs. In the DG class, these strains were dominated by DG(38:1e) and DG (31:2e) and showed no significant quantitative differences (Figure S2). TGs and DGs had fatty acids with two double bonds in contrast to the other lipid classes. TGs with two double bonds were present in low content, whereas DGs with two double bonds (the DG ether) were abundant. The Heysham-1 and T6K-13 strains produced 10 different ceramides and one oxidized ceramide (t20:0_24:0+O). The mutant-type strain contained twice as much Cer(t34:0) and Cer(t38:0) compared with the wild-type strain. The Heysham-1 strain synthesized more Cer(t20:0_26:0), Cer(t18:0_26:0), and Cer(t35:0) than the mutant-type strain (Figure 6).

### 2.4. FLIM-FRET Measurements

The resonance excitation energy transfer approach (often referred to as Förster resonance energy transfer FRET was applied to analyse the interaction of *L. pneumophila* with THP-1-derived macrophages and *A. castellanii* cells. For this purpose, the interacting cells were labeled with fluorescence labels, acting as excitation energy donor and acceptor: *L. pneumophila* with Syto9 (donor) and *A. castellanii* and THP-1-derived macrophages with Nile Blue (acceptor). The fluorescence emission spectrum of the donor and absorption spectrum of the acceptor are presented in Figure S3. An overlap of those spectra (marked in Figure S3) enables the determination of the Förster distance of this donor–acceptor pair (R_0_ = 37 Å, the distance at which the energy transfer efficiency is 50%). Based on R_o_ and measurements of the intensity of fluorescence of the acceptor excited at the wavelengths characteristic of light absorption by the donor, one can evaluate both excitation energy transfer efficiency (E_FRET_) and inter-fluorophore distance (R) (Figure S3).

**Figure 7:**
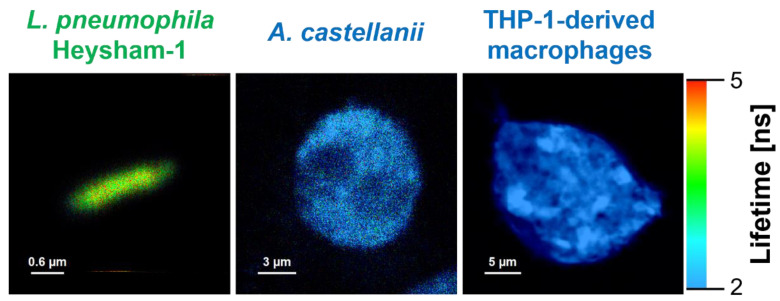
FLIM images of *L. pneumophila* Heysham-1 strain labeled with Syto9 and *A. castellanii* and macrophages labeled with Nile Blue. Two sets of excitations have been applied: 470 nm for *L. pneumophila* Heysham-1 (observation via band pass filter 550/88) and 635 nm for *A. castellanii* and THP-1-derived macrophages (observation via band pass filter 690/70).

**Figure 8:**
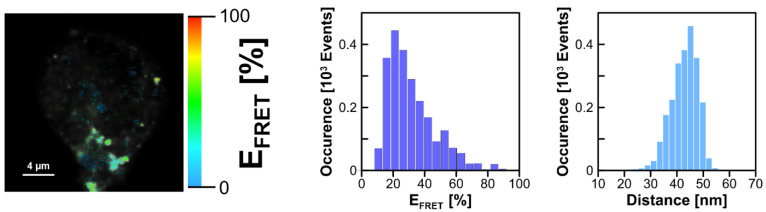
Images illustrating Förster type excitation energy transfer (FRET) between Syto9 immobilized on the surface of *L. pneumophila* cells (Heysham-1) and NB situated within THP-1-derived macrophage. The left panel shows an image based on the efficiency of excitation energy transfer. The middle panel shows a histogram of the distribution of FRET efficiency and the right panel a histogram of the distribution of the calculated donor–acceptor distance. Excitation set at 470 nm.

Figure 7 presents fluorescence lifetime-based images (FLIM) of *L. pneumophila* strains, *A. castellanii*, and THP-1-derived macrophages. Since the bacteria were labeled with Syto9 and the *A. castellanii* and THP-1 derived macrophages with Nile Blue, for imaging purposes, excitation and emission wavelengths have been selected to enable the effective fluorescence imaging of bacterial and eukaryotic cells. In order to analyse the interaction of *L. pneumophila* strains with *A. castellanii* or THP-1-derived macrophages, the excitation wavelength was set to selectively excite Syto9 (470 nm) but fluorescence was observed at wavelengths representing exclusively light emission using Nile Blue (690/70 nm). An example of such an experiment is shown in Figure 8, presenting the image created based on the efficiency of excitation energy transfer, the distribution of E_FRET_, and the distribution of inter-fluorophore distances.

The FRET image shows an effective interaction of *L. pneumophila* strains with the THP-1 macrophage, with most of the E_FRET_ values being higher than 20%.

**Figure 9:**
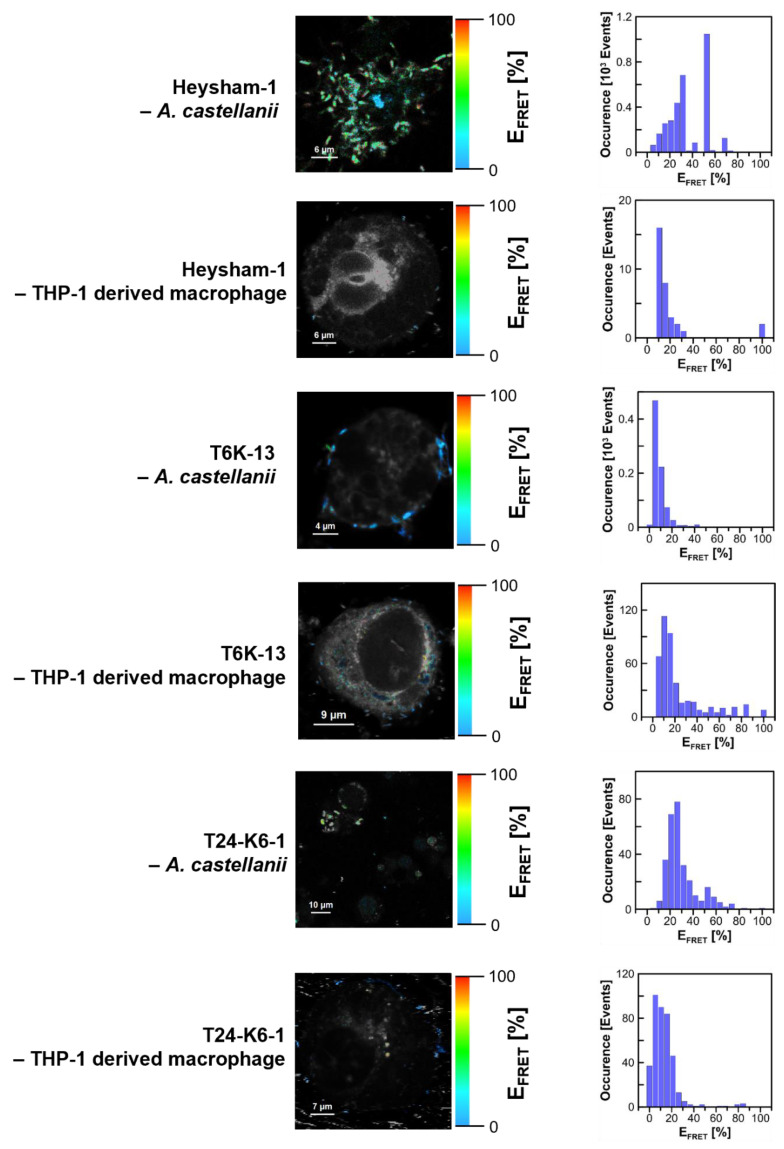
Images illustrating Förster type excitation energy transfer (FRET) between Syto9 immobilized at the surface of *L. pneumophila* cells (Heysham-1, T6-K13, T24-K6-1) and NB situated on *A. castellanii* cells and macrophages. The left panel shows images based on the color-coded efficiency of excitation energy transfer between fluorescence probes situated on *L. pneumophila* and *A. castellanii* or THP-1-derived macrophages (selective excitation of the donor and fluorescence emission of the acceptor). The right panel shows the FRET efficiency histogram representing an analysis of the map shown on the left panel. Excitation set at 470 nm.

**Figure 10:**
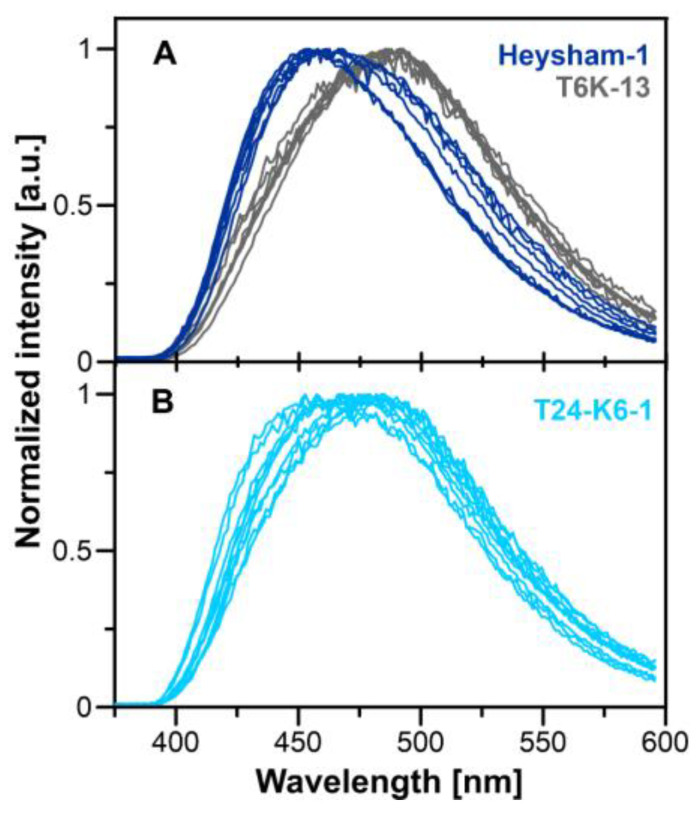
Fluorescence emission spectra of the Prodan molecular probe immobilized at the surface of *L. pneumophila* cells recorded at different locations in the microscopic images of Heysham-1 (dark blue traces), T6K-13 (gray traces) (**A**) and T24-K6-1 (light blue traces) (**B**) strains.

Figure 9 presents the comparison of FRET-based images of the *L. pneumophila* Heysham-1 strain interacting with *A. castellanii* and THP-1-derived macrophage. In addition to the wild-type strain, the T6K-13 mutant-type strain with a defect in LPS synthesis and the T24-K6-1 strain with a restored LPS synthesis pathway were examined. In general, the image analysis shows that the bacteria cells interact effectively and are localized preferably in the surface region of both the THP-1-derived macrophage and *A. castellanii* cell. Interestingly, the FRET efficiency was relatively low in the case of the T6K-13 cells interacting with *A. castellanii*, thus indicating that the presence of LPS promotes the *L. pneumophila* interaction with *A. castellanii*. Importantly, the complementant T24-K6-1 strain also presents FRET events characterized by higher E_FRET_ values (>20%). Such an effect strongly supports the conclusion regarding the promoting effect of LPS. In order to further study possible differences in the surface of *L. pneumophila* associated with the presence of LPS, which can influence interaction with other cells and biomembranes, *L. pneumophila* cells were stained with Prodan fluorescence dye, recognized to be highly sensitive to the direct environment, to assess its physicochemical properties, in particular its polarity (Figure 10).

The application of fluorescence spectroscopy with sub-microscopic spatial resolution enabled the detection of fluorescence emission spectra of the Prodan molecules immobilized in the surface region of single bacterial cells. The fluorescence emission spectra recorded from the *L. pneumophila* Heysham-1 strain, the T6K-13 mutant-type strain, and the complementant T24-K6-1 strain cell surface are presented in Figure 10. As can be seen, a variety of spectra with the maximum shifted along the wavelength axis were recorded. This is a manifestation that the fluorescence label molecules bind to different locations at the cell surface, characterized by more polar (plotted in gray) or more hydrophobic (plotted in dark blue) environments. Interestingly, such spectral shifts are relatively limited in the case of the T6K-13 mutant-type strain cells. In particular, the hypsochromically shifted spectra, representing the location of Prodan molecules in a more hydrophobic environment, were limited in the case of cells with modified LPS. It is very likely that such an effect is indicative of a general modification of the cell membrane and is not a direct representation of the binding of the fluorophore to LPS. As can be seen, in the case of the complementant cells, the shift distribution of the Prodan emission spectra is restored to some extent (light blue, lower panel).

### 2.5. LPS Contributes to Host Cell Adherence and Invasion

**Figure 11:**
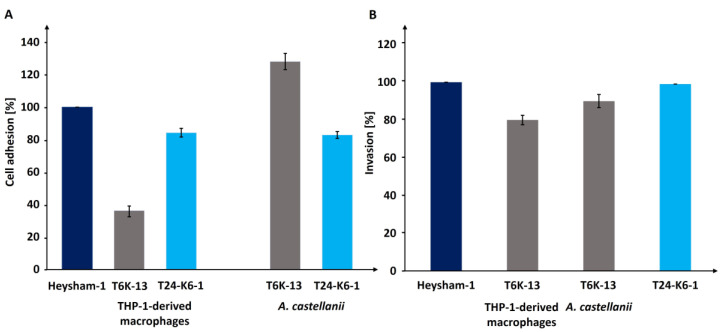
The ability of *L. pneumophila* strains (Heysham-1; T6-K13; T24-K6-1) to adhere to (**A**) and invade (**B**) THP-1 derived macrophages and *A. castellanii*.

The adhesion assay revealed that *L. pneumophila* strains bound to host cells differently. The adherence of the T6K-13 mutant-type to THP-1-derived macrophages was decreased by 64% compared with the *L. pneumophila* wild-type phenotype. Complementation restored the ability of the mutant to adhere like that of the wild-type strain. However, in interaction with *A. castellanii* cells, the T6-K13 mutant-type strain showed a 28% improvement in adhesion compared with the wild type (Figure 11A).

To determine whether the mutation in LPS affects the invasion of eukaryotic cells by *L. pneumophila* strains, the ratio of intracellular bacteria to total cell-associated bacteria was determined using differential immunofluorescence staining. The results showed that the invasion of the T6-K13 mutant-type strain was reduced in both host cell types, with an invasion rate of 80% observed in THP-1-derived macrophages and 90% in *A. castellanii* (Figure 11B).

### 2.6. The T6-K13 Mutant-Type Exhibits a Higher Replication Efficiency within Host Cells than the Wild-Type Strain

**Figure 12:**
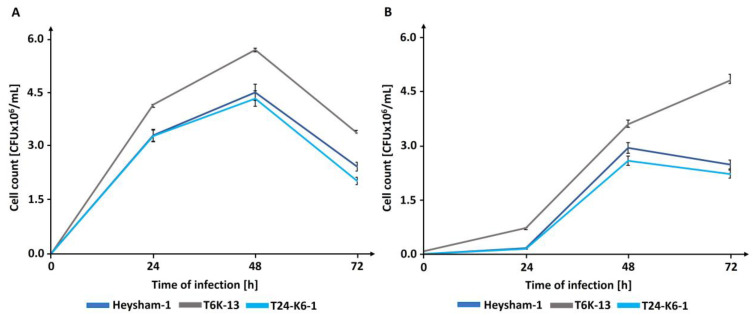
Effect of *L. pneumophila* LPS on the bacterial replication. THP-1-derived macrophages (**A**) or *A. castellanii* cells (**B**) were infected with the wild-type *L. pneumophila* strain Heysham-1, the mutant-type T6K-13 strain, or the complementant T24-K6-1 strain.

To assess the impact of *L. pneumophila* LPS on intracellular replication, THP-1-derived macrophages and *A. castellanii* cells were infected with the wild-type *L. pneumophila* strain Heysham-1, the mutant-type T6K-13 strain, and the complementant T24-K6-1 strain. In both host cell types, the ability of the mutant-type T6K-13 strain to replicate intracellularly was significantly higher than that of the *L. pneumophila* Heysham-1 strain. In THP-1-derived macrophages, the bacterial load of the mutant strain was approximately 25% higher than that of the wild-type strain (Figure 12A). The mutant-type T6K-13 replicated much more effectively in *A. castellanii* cells compared with the Heysham-1 strain, with the greatest difference observed 72 h post-infection when the mutant-type strain load was almost twice as high as that of the wild-type strain (Figure 12B).

### 2.7. The Mutation in the Polysaccharide Region of L. pneumophila LPS Results in a More Potent Induction of Proinflammatory Cytokines

**Figure 13:**
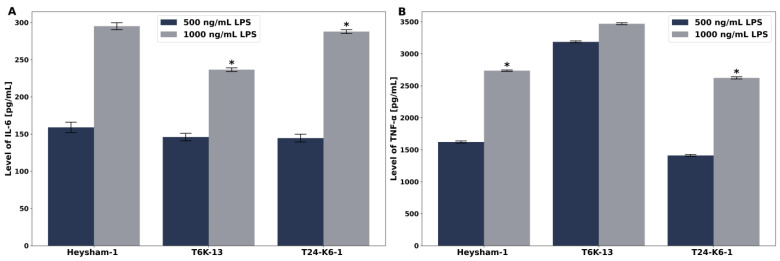
The level of pro-inflammatory cytokines (IL-6) (**A**) and (TNF-α) (**B**) in stimulated THP-1-derived macrophages with LPS preparations of the *L. pneumophila* strain Heysham-1, the T6K-13 mutant strain, and the complementant T24-K6-1 strain. Control IL-6, 4.7 pg/mL; TNF-α, 7.14 pg/mL *—statistically significant difference compared to the control sample (Mann-Whitney U test).

The level of pro-inflammatory cytokine induction (IL-6, TNF-α) was measured in the cell culture supernatant of THP-1-derived macrophages stimulated with LPS preparations at concentrations of 500 and 1000 ng/mL for 4 h (TNF-α) and 24 h (IL-6). *L. pneumophila* LPS preparations induced a measurable level of pro-inflammatory cytokines, which were dose-dependent. The level of induced TNF-α under *L. pneumophila* LPS stimulation was significantly higher compared with the induced level of IL-6. The mutant-type strain proved to be a better inducer of both pro-inflammatory cytokines. The LPS of the mutant-type strain T6K-13 at a concentration of 500 ng/mL induced the production of TNF-α about 2 times and at a concentration of 1000 ng/mL about 1.3 times better compared with the *L. pneumophila* Heysham-1 strain. The level of IL-6 released under the influence of the LPS preparation from the mutant-type strain was slightly lower at a concentration of 500 ng/mL and 1000 ng/mL compared with the level of this cytokine released under the influence of LPS isolated from the *L. pneumophila* Heysham-1 strain (Figure 13A,B).

## 3. Discussion

The approach, attachment, and invasion of host cells (macrophages and protozoan cells) are critical steps in the cellular infection cycle of *L. pneumophila* and if the bacterium fails to execute each of these steps correctly, the number of viable intracellular *L. pneumophila* will be diminished. Numerous factors contribute to these processes, including adhesins, proteins of the type IV secretion system, flagellum, pili, enzymes, and LPS, which facilitate the successful colonization and replication of *L. pneumophila* within host cells, ultimately resulting in the pathogenesis of Legionnaires’ disease [3,24,25,26]. A high-molecular-weight, amphiphilic LPS located in the outer membrane of the cell envelope, forming an extracellular hetero-bilayer with lipids, represents one of the contributing factors.

Our previous studies have shown that the length of legionaminic acid polymer (OPS) and its degree of substitution with *O*-acetyl groups determine the ability to adhere to and interact with *A. castellanii* cells [27]. In the next step, we aimed to investigate whether the core region of the *L. pneumophila* polysaccharide determines the interaction with host cells. For this purpose, a mutant interrupted in the ORF7 gene region of the *L. pneumophila* Heysham-1 strain was constructed. A comparative analysis of NMR spectra of the polysaccharide part from the wild-type strain and the mutant revealed differences in the rhamnose residues of the LPS core region. Although NMR analyses of these samples showed no difference in their degree of acetylation, the mutant-type strain exhibited significantly lower hydrophobicity of its surface compared with the wild-type strain, as confirmed by measuring the degree of polarity using a polarity-sensitive fluorescent dye. This suggests that the mutant is likely lacking an *O*-acetyl group attached to the rhamnose of the core region. The lack of differences in the NMR signals corresponding to the *O*-acetyl group of the core of the wild-type and mutant-type strains may be because the degradation of LPS in the acetate buffer induces the partial migration and partial removal of the *O*-acetyl group from the terminal rhamnosyl group [10].

The presence of *O*-acetyl groups can indirectly influence the control of long-range non-specific electrostatic repulsion experienced by bacteria when approaching negatively charged host cells. The soft surface layer of 8-*O*-acetyl group-bearing mAb3/1 positive *L. pneumophila* strains was significantly less charged and more permeable than those of mAb 3/1 negative strains [28]. Changes in surface charge density can affect the stronger or weaker interaction with eukaryotic cells. The absence of *O*-acetyl groups on rhamnose of *L. pneumophila* LPS may contribute to an increase in the negative surface charge density of bacteria and thus hinder contact with *A. castellanii* cells and macrophages. The proposed mechanism is consistent with the results of excitation energy transfer studies between fluorescent dyes located on the surface of *L. pneumophila* and eukaryotic cells. The Heysham-1 strain exhibited the highest energy transfer efficiency and therefore the strongest interaction, while the T6K-13 mutant showed a weaker interaction. Measurements of the effectiveness of interaction between these bacterial strains and macrophages again indicated stronger interaction with the Heysham-1 strain and weaker interaction with the mutant. However, the interactions of these strains with macrophages were weaker than with amoebae. The next stage of short-range interaction between bacterial and host cells requires ligand–receptor interactions. The attachment of *L. pneumophila* to *A. castellanii* cells may occur through the α1-3d-mannobiose domain of the mannose-binding receptor located on amoebae. An analysis of signals in the NMR spectra of OPS wild-type and mutant strains indicated a slight difference in the range of mannose signals, suggesting that in the case of the mutant-type strain, mannose may be more accessible and therefore the mutant binds better to *A. castellanii* cells. Adhesion tests showed that the mutant-type strain exhibited a 28% increase in adhesion to *A. castellanii* compared with the wild-type strain. These findings demonstrate that eukaryotic cell surface receptors can interact with the intrinsic sugars of the LPS polysaccharide region and mediate the attachment of *L. pneumophila* to these cells. However, the mutant showed weaker binding to macrophages compared with the wild-type strain, indicating that the OPS degree of *O*-acetylation plays a greater role in adhesion to macrophages than to *A. castellanii* cells. Regardless of the infected cell type, the mutant replicated significantly better compared with the wild-type strain, indicating that the LPS structure primarily determines the early stage of host–cell interaction.

The lipid profile of *L. pneumophila* strain Heysham-1 was highly diverse and included phospholipids as the dominant lipid fraction and neutral lipids and ceramides, including oxidized ceramide. *L. pneumophila* synthesizes ceramides, whose fatty acids are mainly even-carbon, saturated, or monounsaturated, similar to those found in eukaryotic cells. Ceramides are rare in bacterial cells, although their presence has been detected in *L. gormanii*. However, *L. pneumophila* does not synthesize hexosylceramides found in *L. gormanii* cells [29]. Mutation in the LPS biosynthesis of *L. pneumophila* also changed the lipid profile of the Heysham-1 strain. These changes affected various lipid groups, but the most significant difference was observed in ceramides. The mutant-type T6-K24-1 synthesized twice as much Cer(t34:0) and Cer(t38:0) compared with the wild-type strain. In Gram-negative bacteria lacking LPS, such as *Sphingomonas* [30] or *Sorangium* [31], sphingolipids functionally replace this outer membrane component. Bacteria possessing both LPS and sphingolipids in response to abiotic stress (acidity or elevated temperature) synthesize more sphingolipids or ceramides, for example, in the cells of *Acetobacter malorum* [32].

Pro-inflammatory cytokines, such as tumor necrosis factor α (TNF-α) and interleukin 6 (IL-6), produced by macrophages, play a crucial role in determining the progression of *L. pneumophila* infection. LPS found in Gram-negative bacteria is one of the most potent immune system stimulators. However, the LPS of *L. pneumophila* is a much weaker cytokine inducer compared with the highly pyrogenic LPS of *Salmonella* Minnesota [33]. The bioactive component of LPS is lipid A, which exhibits toxicity that is directly influenced by the length and number of fatty acid groups attached to its glycosidic backbone. The presence of long-chain fatty acids in the lipid A of *L. pneumophila* leads to its low endotoxic activity, as it fails to interact with the CD14 receptor and its soluble form. Studies on the *L. pneumophila* strain Heysham-1 strain also confirmed the weak ability of LPS to induce cytokines. However, the mutant defective in the synthesis of the polysaccharide part more strongly induced TNF-α, and slightly less IL-6, compared with the wild-type strain. This suggests that the length of fatty acids in lipid A and the structure of the polysaccharide region of LPS determines its ability to induce cytokines.

## 4. Materials and Methods

### 4.1. Chemicals and Reagents

The solvents chloroform, methanol, phenol, and acetic acid were purchased from Avantor Performance Materials Poland (Gliwice, Poland). Ultrapure water, NH_4_formiate, isopropanol, chloroform, acetonitrile, and formic acid were obtained from Merck (Darmstadt, Germany) in LC/MS grade purity.

### 4.2. Bacterial Strains and Growth Conditions

*L. pneumophila* wild-type strain Heysham-1 (ATCC 43107), the mutant-type strain (T6-K13), and the complementant strain (T6-K24-1) were cultured at 37 °C for 3 days on ACES-buffered charcoal-yeast extract (BCYE) agar containing 0.4 mg/mL L-cysteine hydrochloride, 0.25 mg/mL ferric pyrophosphate, and 0.5 mg/mL α-ketoglutarate (Oxoid, Hampshire, UK) [34]. The bacterial mass collected from plates was suspended in 0.5 M NaCl and centrifuged at 8000 rpm for 20 min. The bacterial pellet was washed once with 0.5 M NaCl and once with MQ water. Then, 5 g of lyophilized bacterial mass was used to isolate lipopolysaccharide and 130 mg of lyophilized bacterial mass was used to isolate lipids. The yield of LPS was 1.5% for the Heysham-1 strain and T6-K24-1, and 1.2% for the mutant-type strain. Lipids accounted for 6.8% of the Heysham-1 biomass, 6.6% of the T6-K13 strain, and 7.3% of the complementant strain T6-K24-1.

*L. pneumophila* strains were cultured in N-(2-acetamido)−2-aminoethanesulfonic acid (ACES)-buffered yeast extract broth (BYE) at 37 °C for 24 h. Liquid cultures were used for adhesion, invasion, and infection tests.

### 4.3. Construction of the L. pneumophila Mutant and Complementation

The knock-out mutants of the ‘open reading frame 7’ (ORF 7), annotated as hypothetical protein on the LPS-biosynthesis locus (GenBank: HE980446) of the Heysham-1 strain, were constructed as described before [35,36,37]. In brief, the ORF 7 was inactivated by an insertion cassette containing a kanamycin resistance gene (kanR) using a restriction-free cloning approach and natural transformation of *L. pneumophila*.

The kanR cassette was obtained from the pCDP05 plasmid [38] and purified by amplifying the cassette of the plasmid [Supplementary Material Figure S4].

The insertion cassette consists of the kanR cassette flanked by homologous regions of the ORF 7. This cassette was integrated into the genome of the recipient Heysham-1 strain by natural transformation. The Heysham-1 strain was cultivated on BCYE agar for 72 h. Around 10^8^ cells were used to inoculate BYE broth for an o/n culture. From the o/n culture, 200 µL was used to inoculate 2 mL BYE broth in a plastic round bottom tube (10 mL) which was incubated at 37 °C for 7 h. At an optical density (OD_600_) of 1.0, ~5 ng of purified DNA (MSB spin; Invitek, Berlin, Germany) was added. The DNA was suspended and the tubes were incubated at 30 °C without agitation for 2 days. Then, 500 µL were plated on BCYE plates with kanamycin (25 µg/mL) and incubated at 37 °C for 2 days. Colonies were picked and screened using PCR and Sanger sequencing (primer: Camp ORF 5 mid/Corby 7-9do2rc; [Supplementary Materials Tables S1 and S2].

The sequence-confirmed mutant T6-K13 was screened for an altered LPS structure using monoclonal antibodies of the German National Reference Laboratory for Legionellosis. An indirect ELISA- and immunofluorescence-based screening using the complete mAb-library was performed as described elsewhere [20,37]. Therefore, the strains were cultured for 8 and 24 h to achieve the early exponential growth phase and the post-exponential growth phase.

For the complementation of T6-K13, a plasmid carrying the ORF-7 of Heysham-1 was constructed. Into the pPCR-Script Cam^+^ cloning vector (Agilent, Santa Clara, CA, USA), the purified PCR product (primers LPS ORF 6a uprc/Corby 7–9 uprc) was inserted at the *Srf*I blunt site according to the manufacturer’s instruction leading to the plasmid pMPH7_A. This plasmid was transformed into TOP10 chemical competent *E. coli* cells (Invitrogen, Waltham, MA, USA). Colonies were screened on LB-medium containing ampicillin (100 mg/mL) and chloramphenicol (30 mg/mL). Colonies were screened using the m13 forw/rev primers (Agilent, Santa Clara, CA, USA). Colonies carrying the plasmid pMPH7_A were grown at 37 °C in LB medium and chloramphenicol (30 mg/mL) until the plasmid was finally extracted using the plasmid DNA purification kit QIAprep Spin (Qiagen, Hilden, Germany). The purified pMPH7_A were transformed into electrocompetent T6-K13 cells as described by Shames [39]. The electroporated cells were cultured in liquid media o/n followed by cultivation on BCYE+chloramphenicol (30 mg/mL) o/n for the selection of complemented cells. Colonies were screened using the m13 primer set. Confirmed complementant T24-K6-1 cells were screened for reactivity with mAbs in order to verify a potential regained LPS structure.

### 4.4. Cultivation of Eukaryotic Cells

#### 4.4.1. Culture of *Acanthamoeba castellanii*

Trophozoites of *A. castellanii* (strain ATCC 30234 free of intracellular endosymbionts) were cultured in PYG liquid medium (containing 15 g proteose peptone 3,5 g yeast extract, 10 g glucose, 120 mg NaCl, 3 mg MgCl_2_ × 6H_2_O, 3 mg CaCl_2_, 3 mg FeSO_4_, 142 mg Na_2_HPO_4_, 136 mg KH_2_PO_4_, and water to 1000 mL), at pH 6.6, as described [40]. The culture was kept at 28 °C on a rotary shaker with an acentric rotation of 3 cm (110 rev/min) for 5 days. Trophozoites at 2 × 10^5^ cells/mL density were plated onto 24-well plastic plates (Nunc, Roskilde, Denmark).

#### 4.4.2. Growth and Differentiation of THP-1 Cells

Experiments were performed with the human acute monocytic leukemia cell line THP-1 (ATCC, No TIB-202). THP-1 cells were maintained in RPMI 1640 supplemented with 10% heat-inactivated fetal calf serum (FCS), 10 mM HEPES, 2 mM glutamine, 100 IU/mL penicillin, and 100 µg/mL streptomycin. The cells were seeded in tissue culture plates (Falcon, Bedford, MA, USA) and incubated at 37 °C in a humidified atmosphere of 5% CO_2_. The culture media, antibiotics, and FBS were purchased from Sigma-Aldrich (Steinheim, Germany).

THP-1 cells were seeded onto 24-well plastic plates (Nunc, Roskilde, Denmark) at a density of 5 × 10^5^ cells/well in RPMI 1640 supplemented with 10% FCS, and treated with a final concentration of 50 ng/mL phorbol 12-myristate 13-acetate (PMA) (Sigma-Aldrich, Steinheim, Germany) for three days to induce maturation toward adherent macrophage-like cells [41]. Subsequently, unattached cells were removed, and after three-time washing, adherent THP-1 cells were cultured in the medium without PMA for three consecutive days with daily fresh medium change (without antibiotics). Shortly before the experiments (adhesion, infection, and invasion tests), the cells from extra wells were counted to calculate the number of bacteria used for infection of one THP-1 cell (MOI, multiplicity of infection). The infective dose was determined using the MTT cell viability assay, as described previously [42]. For the FLIM (fluorescence life-time imaging microscopy) analysis, THP-1 cells were cultured on rounded glass slides placed in wells of 6-well plastic plates (Nunc, Roskilde, Denmark) and differentiated according to the above protocol.

### 4.5. In Vitro Cytokine Induction

LPS samples at 500 and 1000 ng/mL were added to the differentiated THP-1 cells. After incubation for 4 h (TNF-α), 24 h (IL-6) at 37 °C, 5% CO_2_, cell culture supernatants were collected and frozen immediately at −80 °C for further cytokines determination. TNF-α and IL-6 levels were measured using the ELISA method using commercial kits from Biorbyt (Cambridge, UK) according to the manufacturer’s instructions. The minimum detectable concentration of TNF-α was 15.6 pg/mL, IL-6—4.69 pg/mL. All experiments were conducted in three independent replicates.

### 4.6. Statistical Analysis

Values were expressed as mean ± S.D. from three independent experiments, each with three independent replicates. Results were statistically evaluated using one-way ANOVA followed by Tukey’s post hoc test and Student t-test (STATISTICA software version 13.3, StatSoft Inc., Tulsa, OK, USA). *p* values of ≤0.05 were considered to be significant.

### 4.7. Lipid Isolation and Ultra-High Performance Liquid Chromatography/Mass Spectrometry (UHPLC-MS) Profiling of Lipids

Lipids were extracted from the dry bacterial mass of *L. pneumophila* strains using a Bligh and Dyer method in which the extraction solvent consisted of chloroform/methanol in a ratio of 1:2 (*v*/*v* [43]. The bacterial mass was suspended in 2 mL MQ water and thoroughly mixed. Next, 7.5 mL of a solvent extraction mixture was added and mixing was maintained for 1 h. Further, 2.5 mL chloroform and 2.5 mL water were added to the suspension, and mixing was continued for 30 min. The sample was centrifuged for 10 min at 3700× *g* and 4 °C. The organic layer (lower) was collected and transferred to a glass bottle. A fresh solvent (2.5 mL of chloroform) was added to the remaining phase. The procedure was repeated four times. The combined organic layers were concentrated in a vacuum evaporator until complete dryness and re-suspended in 4 mL chloroform and 4 mL of a mixture containing 1 mL water and 3.75 mL of chloroform:methanol (1:2 *v*/*v*). The lipid-containing organic phase obtained by centrifugation (as above) was dried in a stream of nitrogen and stored at −20 °C until further processing.

The lipid samples were dissolved in 6 mg/mL CHCl_3_. After vortexing and ultrasonification, centrifugation was performed for 10 min, 14,000 rpm at 4 °C. Then, 100 µL of the supernatant was transferred to an Lc Vial and dried down in a speed vac concentrator. The dried samples were dissolved in 1 mL 20% B with 10% CHCl_3_. Then, 3 µL of each sample was injected into a Vanquish UPLC system (Thermo Scientific, Waltham, MA, USA). Chromatographic separation was performed using a C18 Accucore Polar Premium HPLC column (2.1 × 100 mm, 2.6 µm, Thermo Scientific) with appropriate precolumn at 55 °C. The mobile phase was A: 60% ACN, 10 mM ammonium formate, 0.1% formic acid, and B: 90% isopropanol, 10 mM ammonium formate, 0.1% formic acid in ultrapure water. The flow rate was constant at 0.4 mL/min with a gradient starting at 20% B, increased to 100% in 8 min, held isocratic for 7 min, returned to starting conditions, and equilibrated for 2.5 min. The lipids were detected on a Q exactive plus with HESI ionization (Thermo Scientific) operated in the positive ion mode. MS data were acquired over a scan range of 250–1200 *m*/*z* with full MS resolution of 70,000 and data dependent MS² resolution of 17,500.

Identification and quantification of individual lipid species were performed using LipidSearch 4.2.29 Software from Thermo Scientific (Waltham, MA, USA) on product level (MS/MS fragmentation).

### 4.8. Isolation, Degradation, and Electrodialysis of the Lipopolysaccharide

#### 4.8.1. Extraction of LPS

The bacterial mass was suspended in 50 mM phosphate buffer (pH 7.0) with 5 mM MgCl_2_ and treated sequentially with lysozyme, deoxyribonuclease, ribonuclease, and proteinase K, as previously described [44], and then subjected to LPS isolation using 45% aqueous phenol for 30 min at 68 °C [45]. The crude extract was dialyzed without separation of the layers in distilled tap water for 3 days until free from phenol and 1 day in distilled water. The high molecular weight LPS was purified using ultracentrifugation (105,000× *g*, 4 °C, 4 h), and freeze-dried. Next, LPS was washed with 20 mL of an acetone:chloroform (1:1, *v*/*v*) mixture and dried on air.

#### 4.8.2. Mild Acid Hydrolysis of LPS

LPS samples (Heysham-1 strain 57 mg, T6K-13 38.5 mg, T24-K-6-1 53 mg) were hydrolyzed with 0.1 M acetate buffer, pH 4.4 under a concentration of 10 mg/mL at 100 °C for 4 h. Centrifugation (12,500× *g*, 30 min, 4 °C) was carried out to remove the precipitated lipid A. The supernatant containing the O-polysaccharide was taken into a flask and concentrated in a vacuum evaporator to a volume of approx. 1 mL.

#### 4.8.3. Gel-Permeation Chromatography

The aqueous phase containing O-polysaccharide was fractionated by gel permeation chromatography on a Sephadex G-50 column (6 × 60 cm, Pharmacia, Stockholm, Sweden) using water as eluent to afford a polysaccharide fraction (PS). The elution was monitored using the phenol–sulfuric acid assay of fractions. The yield of the high molecular weight OPS preparation was 26% for the Heysham-1 strain and T6-K13, and 28% for T24-K-6-1 of the LPS mass subjected to hydrolysis.

#### 4.8.4. Electrodialysis

To remove cations, lipopolysaccharides (c. 10 mg) were electrodialysis for 24 h (500 mA, 200 V, 40 W) using Bio-Rad (Hercules, CA, USA) electrophoresis apparatus and lyophilized [46].

### 4.9. NMR Spectroscopy

The LPS samples of *L. pneumophila* were dissolved in 1 mL of 99.0% D_2_O, frozen, and lyophilized. The process was repeated twice, and finally, the samples were dissolved in 650 µL of 99.95% D_2_O. The 1D (1H) and 2D (homonuclear COSY, TOCSY, and ROESY and heteronuclear HSQC and HMBC) NMR spectra were recorded on a Bruker Avance III 700 MHz spectrometer at 39 °C. The mixing time of the ROESY and TOCSY experiments were 0.2, and 0.1 s, receptively. An internal standard of acetone was used to calibrate the chemical shifts (δ_H_ 2.225; δ_C_ 31.45).

All spectra for de-*O*-acetylated samples (12% ammonia, 60 °C, 3 h) were recorded at 39 °C.

### 4.10. Infection Assays with THP-1 Macrophages and A. castellanii

Differentiated THP-1 derived macrophages (2 × 10^5^ cells/mL) and *A. castellanii* (2 × 10^5^ cells/mL) were infected with *L. pneumophila* strains (Heysham-1 strain, T6-K13, T24-K-6-1) from the early stationary phase with a MOI 20 for THP-1 macrophages and *A. castellanii* cells for 2 h at 35 °C. Next, cells were washed twice with PBS to remove non-adherent bacteria, and gentamicin (100 μg/mL) for 1 h was added to kill bacteria that were not phagocytized by eukaryotic cells. No viable bacteria were detected using direct plating of cell culture supernatants on BCYE agar. The experiment included a one-time exchange of the RPMI 1640 culture fluid supplemented with 10% calf serum, without the addition of antibiotics and liquid PYG medium on each day of the experiment in cultures infected with THP-1-derived macrophages and *A. castellanii* cells, respectively. At the indicated time points (0 h, 24 h, 48 h, 72 h), cells were lysed with 0.01% Triton X-100 (Merck, Darmstadt, Germany), and then plated on BCYE medium. The infection efficiency was monitored by determining the number of colony-forming units (CFU) of the different *L. pneumophila* strains after plating on BCYE agar and incubation at 37 °C for 3 days. Three biological replicates of infected macrophages or *A. castellanii* were performed at each time point.

### 4.11. Adhesin Test

THP-1 differentiated macrophages or *A. castellanii* at a density of 2 × 10^5^ cells/mL seeded in 24 well plates were pre-incubated with 10 μM cytochalasin B (Merck, Darmstadt, Germany) for 2 h at 35 °C to block phagocytosis. After this time, *L. pneumophila* strains at a MOI 20 were added to the macrophages or amoeba cells. The infections were synchronized through centrifugation at 300× *g* for 5 min. After 30 min of co-incubation with THP-1 derived macrophages and *A. castellanii* cells, the monolayers were washed twice with PBS to remove non-adherent bacteria and lysed with 0.01% Triton X-100. Serial dilutions of the inoculum and of *L. pneumophila* strains recovered from lysed cells were plated on BCYE agar. The number of adherent bacteria was enumerated after incubation of BCYE plates for three days at 37 °C. The results were expressed as the ratio of adherent bacteria compared with the inoculum. The adhesion test was performed in three independent experiments.

### 4.12. Invasion Assay

Before being added to the eukaryotic cells, *L. pneumophila* strains were washed three times by centrifugation (6000× *g* for 5 min) and stained with 0.3 mM rhodamine B for 25 min (Merck, Darmstadt, Germany). The eukaryotic cells at a density of 2 × 10^5^ cells/mL were infected for 1 h with rhodamine-stained *L. pneumophila* strains. After washing the unbound bacteria twice in PBS, the cells were fixed with 4% PFA overnight at 4 °C. PFA was then removed by washing with PBS containing 50 mM glycin, and the samples were blocked by 10% NHS in SorC-buffer. The extracellular bacteria were labeled with a polyclonal rabbit α-*L. pneumophila* antibody (ab20943) followed by an Alexa Fluor^®^ 488-coupled goat α-rabbit antibody (ab150077) (Abcam, Cambrigde, UK).

The process of invasion of THP-1-derived macrophages and *A. castellanii* cells by *L. pneumophila* strains was observed in a confocal laser scanning microscope (Leica DM4000 B, Wetzlar, Germany). For each strain, at least 50 cells with associated bacteria were analysed in three independent experiments, and the ratio of internalized bacteria was calculated.

### 4.13. Steady-State Spectroscopy

Stationary measurements of the absorption and emission spectra of the donor (Syto9) and acceptor (Nile Blue) energy were performed on a Cary 60 UV-VIS spectrometer and a Cary Eclipse spectrofluorometer, respectively (both from Agilent Technologies, Santa Clara, CA, USA). Based on the recorded spectra of dyes dissolved in physiological fluid, the Förster distance was calculated according to the formula:

$$R_{0}=8.79\times 10^{3}{\left[Q_{D}\kappa^{2}n^{-4}J\left(\lambda \right)\right]}^{-\frac{1}{6}}$$

where *Q_D_* is the fluorescence quantum yield of donor (*Q_D_* = 0.58), *κ^2^* is an orientation factor (here 0.476), *n* is the refractive index of the medium (*n* = 1.33), and *J*(*λ*) is the overlap integral:

$$J\left(\lambda \right)=\frac{\int F_{D}\left(\lambda \right)\varepsilon_{A}\left(\lambda \right)\lambda^{4}d\lambda }{\int F_{D}\left(\lambda \right)d\lambda }$$

where *F_D_*(*λ*) denotes donor fluorescence intensity, *ε_A_*(*λ*) is the extinction coefficient of acceptor, and *λ* is a wavelength. Taking into account the molar decadic extinction coefficient for Nile Blue 18,000 M^−1^cm^−1^, the Förster distance (*R*_0_) of Syto9-Nile Blue donor–acceptor pair is 37 Å.

#### 4.13.1. Microscopy Imaging

For the FLIM analysis, *L. pneumophila* cells (Heysham-1, T6K-13, T24-K-6-1) (100 µL of suspension each, OD_600_ = 0.2) in MQ water were incubated with 5 µM Syto9 green fluorescence stain in 50 mM Tris pH 7.0 buffer at 20 °C for up to 20 min (Thermo Fisher Scientific, Waltham, MA, USA). After centrifugation at 5000× *g* for 5 min at 22 °C, the bacteria were washed 3 times and resuspended in 100 µL of non-pyrogenic water. Then, 10 µL of Syto9-labeled bacterial suspension was added to Nile Blue (3 µM)-stained *A. castellanii* or macrophages on rounded glass slides and analysed using FLIM microscopy.

The research was carried out on the MicroTime 200 microscopic system from PicoQuant GmbH (Berlin, Germany) enabling the simultaneous measurement of the fluorescence intensity and lifetime. The system was based on the Olympus IX71 microscope (Tokyo, Japan). After excitation with laser light with a wavelength at 470 nm or 635 nm, the fluorescence emission signal was collected using silicone immersed oil objective (Olympus, Tokyo, Japan) with a magnification of 60× and a numerical aperture of 1.3. The selection of these excitation wavelengths was determined by the selection of fluorescent markers serving as energy donor (Syto9) and acceptor (Nile Blue). The scattered light was removed first with a dichroic mirror ZT470/488/640/RPC (AHF analysentechnik, Tübingen, Germany), then, after separation with another dichroic mirror 620 dcxxr (AHF analysentechnik, Tübingen, Germany), was directed to two detectors in the form of avalanche photodiodes SPCM-AQRH-TR (Excelitas, Waltham, MA, USA). Prior to each of the detectors, the fluorescent signals were once again filtered for the donor and acceptor channels, respectively, by a 550/88 bandpass filter (Semrock, Rochester, NY, USA) and 690/70 bandpass filter (AHF Analysentechnik, Tübingen, Germany). The microscope was operated in the confocal mode with a pinhole diameter of 50 µm. The microscope scanned selected areas of studied objects with a constant resolution of 200 × 200 pixels. The dwell time which provided an excellent signal-to-noise ratio for each pixel was set to 0.4 ms. In the measurements, pulsed light was used with a frequency of 20 MHz and intensity of ~50 nW for both lasers. The analysis of the obtained results was performed with the use of dedicated software SymPhoTime v. 2.6 by PicoQuant GmbH (Germany).

The efficiency of the FRET process (*E_FRET_*) has been calculated for every pixel of the collected microscopic images and properly calculated from the formula:

$$E_{FRET}=\frac{I_{A}}{I_{A}+\gamma I_{D}}\times 100\%$$

where *I_D_*, *I_A_—*donor and acceptor intensity (background level corrected), respectively, *γ—*compensation for detector detection efficiency between donor and acceptor channel.

The actual distance (*R*) between the interacting dyes has been calculated from the formula:

$$R=R_{0}{\left(\frac{1}{E}-1\right)}^{\frac{1}{6}}$$

#### 4.13.2. Microscopy Spectra

*L. pneumophila* cells (Heysham-1, T6K-13, T24-K-6-1) (100 µL of suspension each, OD600 = 0.2) in MQ water were incubated with 0.1 µM prodan at 22 °C for up to 5 min. The microscopic spectra of prodan labeling bacteria were acquired using a spectrograph SR-163 equipped with a Newton 970 EMCCD camera from Andor Technology (Concord, MA, USA) connected to a MicroTime 200 system. For this purpose, excitation at 375 nm and long-pass filter FF01-380LP (Semrock, Rochester, NY, USA) were used. The microscope, similarly to the FRET measurements, worked in the confocal mode. Spectra were collected for several bacteria immobilized on a coverslip using polylysine.

## 5. Conclusions

The mutant interrupted in the ORF7 region of the *L. pneumophila* strain Heysham-1 LPS biosynthetic gene is likely lacking the *O*-acetyl groups attached to the rhamnose of the OPS core. The absence of these *O*-acetyl groups alters the epitope recognized by the monoclonal antibodies of the Dresden Panel. The *O*-acetyl groups of the core region determine the interaction with *A. castellanii* cells and macrophages at an early stage of establishing contact with host cells. They also contribute to stronger adhesion to macrophage cells. On the other hand, the lack of core *O*-acetyl groups contributed to the exposure of the mannose receptor and the better binding of these bacteria to *A*. *castellanii* cells. This means that the initial cross-talk between *L. pneumophila* and *A. castellanii* differs from that between *L. pneumophila* and macrophages. The mutant multiplied much better in both amoeba and macrophage cells, indicating that the *O*-acetyl groups of the core region determine the early stages of interaction with host cells before entering the target cell. Mutation in the LPS biosynthesis of *L. pneumophila* also resulted in a change in the lipid profile. These changes affected various lipid groups, but the most significant difference was observed in ceramides. The mutant more strongly induced TNF-α, compared with the wild-type strain. This suggests that the structure of the polysaccharide region of LPS as well as PS decorations with non-sugar substituents determines its ability to induce cytokines. The modulation of the O-antigen structure may be one of the mechanisms employed by *Legionella* bacteria to evade the control of the host’s immune system.

## Figures and Tables

**Table 1:** ^1^H and ^13^C NMR 700 MHz data of the OPS isolated from the mutant T6-K13.

Residue	Chemical Shifts (ppm) ^1^H and ^13^C								
	H1 C1	H2 C2	H3 C3	H4 C4	H5 C5	H6 C6	H7 C7	H8 C8	H9 C9
→ 4)-α-Leg*p*-(2 →	- 174.36	- 101.87	2.635/1.630 39.51	4.057 72.06	3.529 54.46	3.994 72.72	3.913 55.43	3.920 67.91	1.202 19.72
*N*-Acetyl	- 175.24	2.103 23.29							
*N*-Acetimidoyl	- 167.73	2.264 20.11							

## Data Availability

Not applicable.

## Supplementary Materials

The following supporting information can be downloaded at: https://www.mdpi.com/article/10.3390/ijms241914602/s1.
