# Supplementary material for: Lipopolysaccharide of Legionella pneumophila Serogroup 1 Facilitates Interaction with Host Cells
Source: Int J Mol Sci. 2023 Sep 27;24(19):14602. doi: 10.3390/ijms241914602 (PMC10572746; doi:10.3390/ijms241914602)
Supplement: Supplementary file 1 [file ijms-24-14602-s001.zip › ijms-2597354-supplementary.pdf]

Supplementary Materials

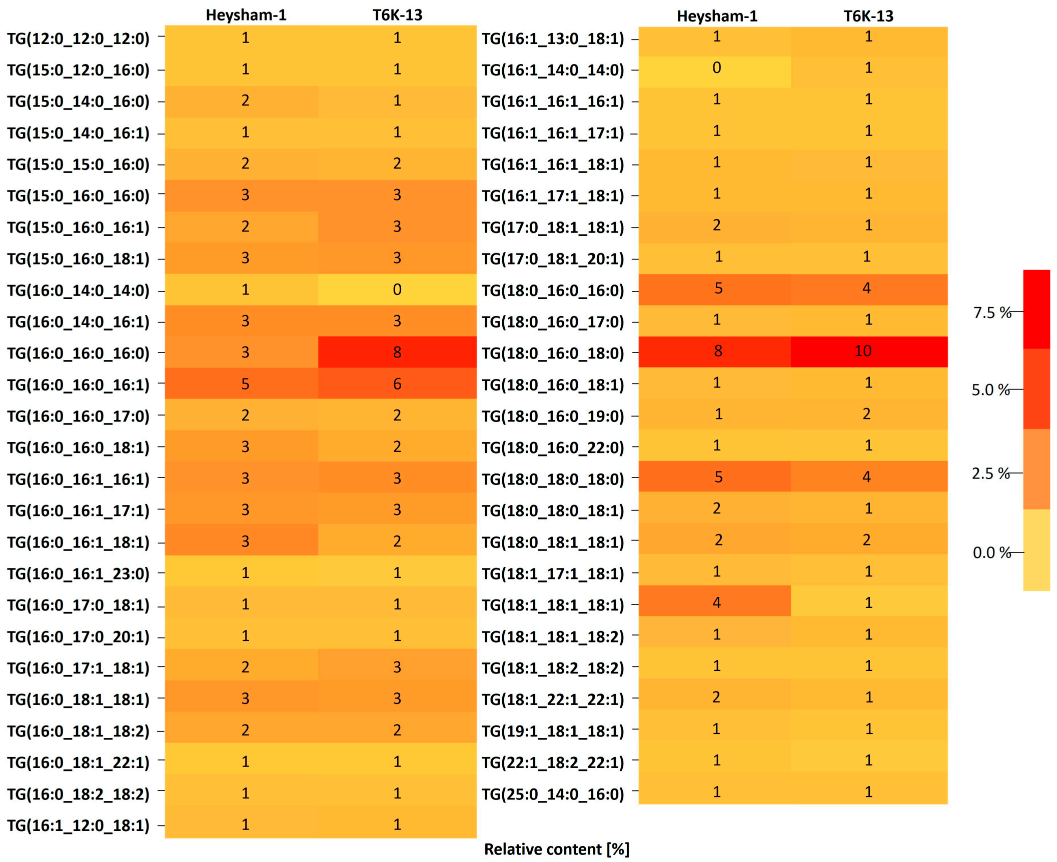

Figure S1. Heatmap visualization of the main species of triglycerides (TG) isolated from the Heysham-1 strain and the T6-K13 strain. TGs were analysed by LC-MS/MS in the positive ion mode.

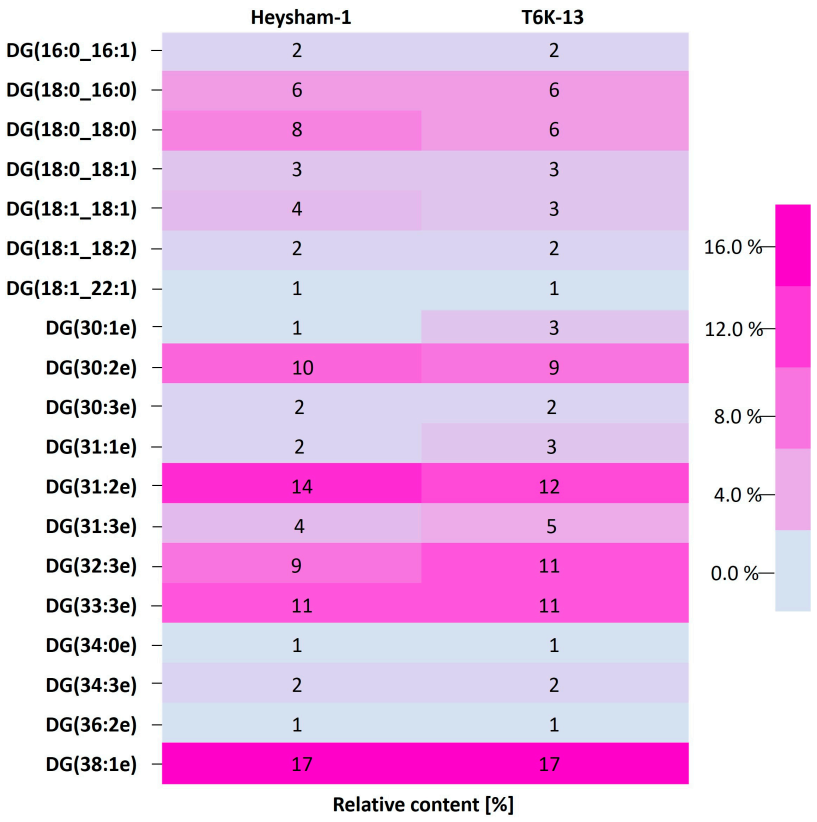

Figure S2. Relative abundance of the main diglycerides (DG) species of the *L. pneumophila* Heysham-1 strain and the T6-K13 strain. DGs were analysed by LC-MS/MS in the positive ion mode.

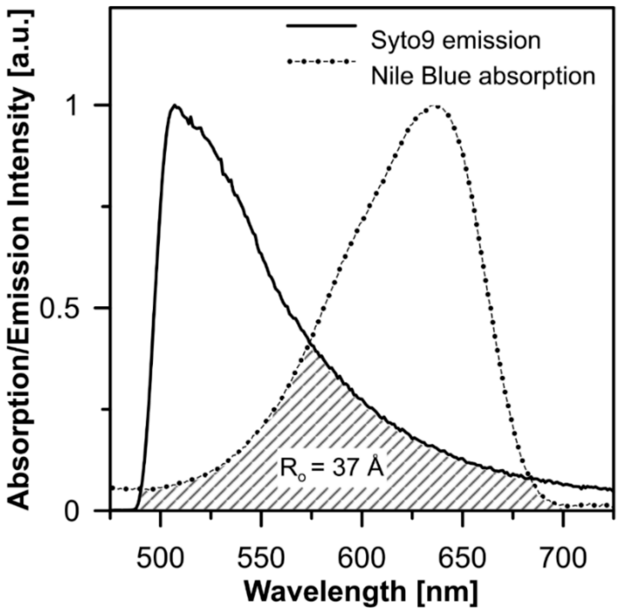

Figure S3. Normalized absorption of NB (dotted line) and emission spectra of Syto9 (continuous line). Emission recorded with 470 nm excitation laser. The spectral overlap (marked) has been used to calculate the Förster distance of this donor-acceptor pair R_o.

Restriction free cloning:

The procedure of the restriction free cloning is based on the amplification of the reporter gene (here Km^R-cassette) which is annealed to flanking regions of the insertion region (here ORF 7 of the LPS-biosynthesis locus of the *L. pneumophila* strain Heysham-1; supplemented material figure 1). Three PCRs amplify the upstream flanking region (PCR A), the reporter gene (PCR B) and the downstream flanking region (PCR C). A next step is the annealing and amplification of PCR A and PCR B followed by the annealing of PCR A&B with PCR C. The primers have an overlap of 15-25 bp with the adjacent regions. The PCR products were controlled by size on a gel and purified using the MSB spin kit (Invitek, Germany). Additionally, the PCR products were sequenced using Sanger sequencing at the local sequencing unit (Max Planck Institute of Molecular Cell Biology and Genetics, Dresden).

Figure S4

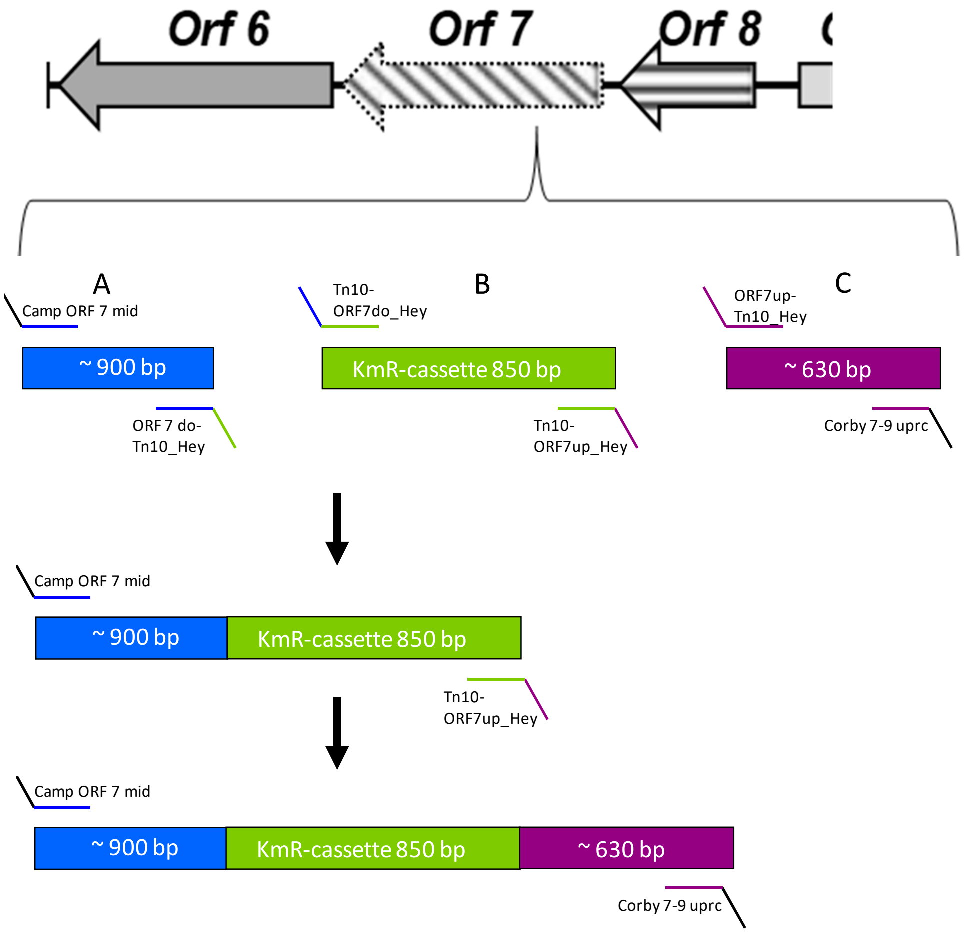

Table S1. Primers used for PCR amplification of the insertion cassette

PCR A: Upstream region (ORF 7)
Primer Camp ORF 7 mid
Sense: 5′-
Primer ORF7do-Tn10_Hey
Antisense: 5′- CGATGAGTTTTTCTAATCAGAATTGTAAGTGCTAGCTTACATAATGTTCC – 3′
PCR B: Kanamycin resistance cassette
Primer Tn10-ORF7do_Hey
Sense: 5′ - GGAACATTATGTAAGCTAGCACTTACAATTCTGATTAGAAAAACTCATCG - 3′
Primer Tn10-ORF7up_Hey
Antisense: 5′ - GCAGTTCTGGATCAAATAGATAATTCAAGGGGTGTTATGAGCCATATTCA- 3′
PCR C: downstream region (ORF 7)
Primer ORF7up-Tn10_Hey
Sense: 5′ – TGAATATGGCTCATAACACCCCTTGAATTATCTATTTGATCCAGAACTGC – 3′
Primer Corby 7-9 uprc
Antisense:

Table S2. The PCRs were performed using the following protocol:

Reaction volume	50 µL
Primer 1	1 µL (10 pmol/reaction)
Primer 2	1 µL (10 pmol/reaction)
dNTP’s	1 µL (10 nmol/reaction)
GoTaq polymerase (Promega, Germany)	0,5 µL (2,5 U/reaction)
DNA	1 µL (100-300 nmol/reaction)
PCR grade H_2O	45.5 µL

After an initial denaturation step at 95 °C for 3 min 30 cycles followed with 95 °C for 30 s, 3.5 °C for 40 s and an elongation step at 72 °C for ~1 min/kb expected PCR product. A final elongation step for 5 min finalized the amplification procedure.
